# Alcohol consumption and alcohol policy

**DOI:** 10.3906/sag-2002-237

**Published:** 2020-08-26

**Authors:** Mustafa Necmi İLHAN, Dilek YAPAR

**Affiliations:** 1 Department of Public Health, Faculty of Medicine, Gazi University, Ankara Turkey

**Keywords:** Alcohol consumption, policies, public health

## Abstract

Alcohol is a unique addictive substance used by many people for different reasons. Alcohol use affects not only the users but also the family and the whole society in a negative way. Alcohol is one of the most commonly used substances for entertainment purposes in the world and 1 in 3 people is a current drinker. 2348 billion people (43% of the population) aged 15 and over are current drinkers and males drink about 2 times more frequently than females (53.6%/32.3%). According to the Global Alcohol and Health Report published by the World Health Organization (WHO) (2018), total alcohol per capita consumption (APC) worldwide aged 15 and over increased from 5.7 liters in 2000 to 6.4 liters in 2016. Harmful alcohol consumption is a major public health problem and it is known to be associated with more than 200 diseases and injuries. Policies and measures to prevent alcohol use are not implemented adequately and the burden of alcohol-related illnesses continues to increase tremendously. In order to prevent and reduce the harmful effects of alcohol, alcohol policies should be formulated based on the best evidence from a public health perspective.

## 1. Introduction

Alcohol, chemically termed ethanol, is a natural, enjoyable substance that causes intoxication while also having a sedative, suppressing brain function [1]. Alcohol is a unique addictive substance used by many people for different reasons. Although many people have emphasized the harm of alcohol on health since Hippocrates, it has only started to be evaluated as a disease in the last hundred years [2]. Alcohol use affects not only the users but also the family and the whole society in a negative way. Besides, the use of alcohol and other addictive substances among individuals who commit crimes and violations of the law shows that the use of any substance is an important social problem [3,4]. In the globalizing world, with the positive support of the media and television, the fact that the actors in movies drink alcohol and light a cigarette creates a desire for people to use these 2 substances.

## 2. Alcohol consumption

Problems related to alcohol use are considered as a public health problem ranging from social drinking, problematic drinking and risky drinking to alcohol addiction. Alcohol is one of the most commonly used substances for entertainment purposes in the world and 1 in 3 people is a current drinker. 2348 billion people (43% of the population) aged 15 and over are current drinkers and males drink about 2 times more frequently than females (53.6%/32.3%). As shown by the most recent WHO data, the prevalence of alcohol consumption decreased from 47.6% in 2000 to 43.2% in 2016. This decrease in the frequency of alcohol use, unfortunately, did not follow the same acceleration as the total APC. According to the Global Alcohol and Health Report published by the WHO (2018), total APC worldwide aged 15 and over increased from 5.7 liters in 2000 to 6.4 liters in 2016. Unrecorded consumption accounts for 26% of the worldwide total consumption [2]. The highest levels of per capita alcohol consumption and alcohol use disorders are observed in countries of the WHO European Region, while the lowest alcohol consumption (2.9% of the population) is observed in the Eastern Mediterranean Region [5,6]. Alcohol use is a complex social problem affecting health. Alcohol is usually consumed before, together or after the use of other psychoactive substances. Although alcohol consumption plays an important social role in many cultures, most countries try to limit or control alcohol use through laws governing the production, sale, and consumption of alcoholic beverages. Total alcohol per capita consumption in the population of Turkey was observed 2.0 liters of pure alcohol in 2016. This amount is much lower than in European countries. The population who did not use alcohol participated in the calculation of average individual consumption. Therefore, misleading conclusions are reached about the amount of use of alcohol. This situation causes alcohol to be ignored as a risk factor in our country and similar countries. Additionally, it is not known how many of the changes in consumption over the years are due to the change in personal consumption or the change in the number of new users [7]. In the years 2010–2016, the results of national health surveys conducted in Turkey show that 10.4–14.9% of the population aged 15 and over uses alcoholic beverages [8]. The amount of alcohol consumption varies among countries. These differences are due to complex reasons such as socio-demographic factors, culture, and economic development.

The effect of alcohol consumption on illness and injury is related to 2 different dimensions of alcohol consumption. The first is the total amount of alcohol consumed and the second is the form of alcohol use. One of the risky situations in alcohol use is heavy alcohol consumption defined as the use of pure alcohol over 60 g. Diseases associated with alcohol use have been associated with heavy episodic drinking [9]. Heavy episodic drinking is another indicator of serious alcohol consumption which WHO defines as at least once a month and at least once 60 g or more of pure alcohol (6+ standard drinks) [5,9]. In the Russian Federation, some other European countries (e.g., Bulgaria, Poland, and Romania) and in some sub-Saharan African countries (e.g., Angola, Democratic Republic, and Congo), heavy episodic drinking is very high among drinkers (≥60% of current drinkers). In other sub-Saharan countries, Australia and some countries in South America (e.g., Bolivia, Brazil, Paraguay, and Peru), heavy episodic drinking occurs at high rates (45–60%). Worldwide, the frequency of heavy episodic drinking in the total population decreased from 22.6% in 2000 to 18.2% in 2016 [2].

Harmful alcohol consumption is an important public health problem associated with more than 200 diseases and injuries. Health problems or conditions with which it is associated; neuropsychiatric disorders, cardiovascular diseases, liver diseases, various cancers, infectious diseases such as HIV/AIDS, traffic accidents, violence, suicide, broken family, and early death [10]. Alcohol affects sensory and motor skills by reducing the reaction time with acute toxic effects and leads to accidents, violent tendencies, suicides, and alcohol poisoning [11]. Alcohol is the third major risk factor for disease burden. Worldwide 3.3 million people die from harmful alcohol use and alcohol-related deaths account for 5.3% of all deaths. Harmful use of alcohol has more impact on mortality than diabetes (2.8%), digestive system diseases (4.5%), traffic accidents (2.5%), tuberculosis (2.3%), HIV/AIDS (1.8%), and hypertension (1.6%). In 2016, alcohol consumption caused 5.1% of all the disability-adjusted life years (DALYs) [5]. The harmful use of alcohol is directly or indirectly related to many health related targets of the Sustainable Development Goals (SDGs 2030). SDGs consist of 17 goals and 169 targets including maternal and child health, infectious diseases (HIV, viral hepatitis and tuberculosis), noncommunicable diseases and mental health, injuries, and intoxications. Alcohol per capita consumption per year in liters of pure alcohol is 1 of 2 indicators for SDG Health Target 3.5-“Strengthen the prevention and treatment of substance abuse, including narcotic drug abuse and harmful use of alcohol” [12].

## 3. Factors affecting alcohol consumption

Multiple factors have an impact on alcohol consumption and alcohol-related diseases and injuries [11]. These factors can be categorized into 2 main headings: social and individual factors (Table 1).

**Table 1:** Factors affecting alcohol consumption.

Social factors	Individual factors
Economic status	Age
Cultural reasons	Sex
Environmental reasons	Familial factors
Alcohol production	Race/ethnicity
Alcohol distribution and related regulations	Socioeconomic level

### 3.1. Age

It is stated that the age of onset for alcohol use goes down to 10 years of age [13]. The results of school surveys indicate that alcohol use starts early and before the age of 15 in many countries [14,15]. Young people (mostly men) often consider drinking as an indicator of adulthood. The drinking behavior of young people reflects the drinking behavior of the society in which they live. For example, according to WHO, the prevalence of current drinking is the highest in the European Region (59.9%), followed by the Region of the Americas (54.1%). Similarly, the prevalence of drinking is still the highest in the European Region (43.8%), followed by the Region of the Americas (38.2%). More than a quarter of the 15–19 age group (26.5%) is still using alcohol worldwide. According to this rate, approximately 155 million adolescents are currently using alcohol. Also, 64.2 million (11%) adolescents stated that they used alcohol but not in the last 12 months. Although the frequency of alcohol use among the 15–19 age group is less than the general population over the age of 15, it is noteworthy that this difference is small and decreases rapidly. The 20–24 age group was reported to be a current drinker with a similar frequency (40.7%) as the general population, and even more frequent in some regions than the general population [5]. According to the results of the European school survey project on the use of alcohol and other drugs, the frequency of alcohol and other drug use of the students were questioned in the last 30 days and it was observed that the frequency of use varies between 50–70% in many countries in America and Europe [14].

### 3.2. Sex

It has been shown in almost all studies that men are at more risk in terms of alcohol use and addiction [5,9,16,17]. According to WHO 2018 data, more than half of (54.6%) women aged 15 years and over never used alcohol at all. As for males, 34.5% also never used alcohol. Besides, it was stated that the amount of alcohol consumed by women and the frequency of heavy episodic drinking were less than those of men. In the adolescent age group, men’s both alcohol consumption and frequency of use were higher [5]. According to WHO reports, the prevalence of alcohol use decreased from 57.9% to 53.6% in males and from 37.3% to 32.3% in women in 2000.

### 3.3. Economic status

There is a positive correlation between the economic level of the country and alcohol consumption. In high-income countries, alcohol consumption per capita is 9.8 liters and only 11.4% of all alcohol consumption is unregistered. In low- and middle-income countries, the amount of alcohol consumption per capita is 3.8 and 4.7 liters, respectively. About 40% of all alcohol consumed in these countries is unregistered. It is estimated that alcohol consumption per capita will increase in people over 15 years of age until 2025, especially in America and Southeast Asia. Unless the increase in alcohol consumption in these regions stops, per capita alcohol consumption is expected to be 6.6 liters in 2020 and 7 liters in 2025 [5]. The 2016 age-standardized burden of deaths was highest in lower-middle-income countries (46.2 deaths per 100,000 people) and low-income countries (42.1 deaths per 100,000 people). Observational studies have identified a socioeconomic gradient among risk factors that are related to alcohol consumption including smoking, fruit and vegetable intake, obesity and physical inactivity; however, these studies originate from developed countries and results may not be the same in lower-income countries [18–20].

## 4. Alcohol policy and interventions

Alcohol control policies are laws, rules, and regulations aimed at preventing and reducing alcohol-related damages. Alcohol policies can be a global, regional, international or national level. Effective alcohol control strategies include a multi-component approach such as access, price, marketing, and drink-driving. WHO’s 5-year strategic plan, the 13th General Program of Work 2019–2023 (GPW13), note that actions to reduce the harmful use of alcohol is a global priority. WHO published SAFER, a new initiative and technical package outlining 5 high-impact strategies that can help governments to reduce the harmful use of alcohol and related health, social, and economic consequences (Figure) [21]. The WHO led initiative package aimed to support the goal of reducing the harmful use of alcohol by 10% by 2025.

WHO member states are required to lead and raise awareness of the burden of diseases related to alcohol use in a meaningful and sustainable way to reduce the harm caused by alcohol consumption. Some countries impose a ban on alcohol in order to eliminate the purchase or consumption of alcohol. The Global Alcohol and Health Survey 2016 shows that 11 countries have an alcohol ban [5]. Table 2 summarizes WHO’s recommended alcohol policies in the Global Alcohol and Health Report 2018.

**Figure:**
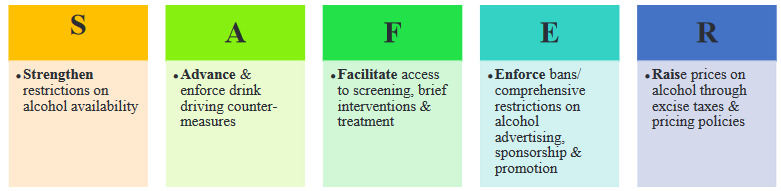
Meaning of SAFER.

**Table 2:** Alcohol policy and interventions.

Leadership, awareness, and commitment · Written national policies · Nationwide awareness-raising activities	Regulating the availability of alcohol · National control of production and sale of alcohol · Restrictions on on-premise and off-premise sales of alcoholic beverages · National minimum age for purchase · Restrictions on drinking in public.
Health services’ responses	
Community action	
Pricing policies	
Drink-driving countermeasures · Blood alcohol concentration limits · Drink-driving prevention measures · Drink-driving penalties	
	Marketing restrictions · Restrictions on alcohol advertising · Regulations on alcohol product placement · Regulation of alcohol sales promotions · Methods of detecting infringements of marketing restrictions
Monitoring and surveillance · National surveys on alcohol consumption · Legal definition of alcoholic beverages · National monitoring systems	
	Addressing informal and illicit production · Inclusion of informal or illicit production in national alcohol policies · Methods used to track informal or illicit alcohol
Reducing the negative consequences of drinking · Responsible beverage service (RBS) training · Labels on alcohol containers	

WHO has collected leadership, awareness and commitment indicators on alcohol use under 2 important headings: development of national written alcohol policies and awareness-raising activities. The existence of a written national alcohol policy is the most important and effective indicator of a country’s determination to reduce alcohol-related harm. According to the 2016 Global Alcohol and Health Survey, 80 countries have national written alcohol policies, of which 67% (34 countries) are high-income countries. Most countries in America and Africa do not have national written alcohol policies. Most countries with national written alcohol policies have passed these policies through the government (59%) or national parliamentary approval (28%) [5]. Until recently, the Russian Federation had one of the riskiest patterns of drinking models in the world. According to the 2011 Global Alcohol and Health Report, Russia’s alcohol consumption per capita reached 15.8 liters, which is the fourth highest consumption in the European region [22]. In order to combat these, Russia has implemented a series of evidence-based effective national alcohol policies in 2016 to reduce per capita consumption to 11.7 liters. Awareness of alcohol-related harm in the country may indicate the need for policy changes and new policies. Therefore, awareness-raising activities are the foundation for national written policies [23]. Awareness-raising activities have been mostly related to drink-driving, the young population and the effects of alcohol on health so far.

Health care is a key partner for individual-level interventions to individuals and families at risk or affected by alcohol use. The health sector plays an effective role in raising awareness of alcohol-related harms, supporting alcohol interventions and advocating community support. Health care should provide support to individuals and families at risk or affected by alcohol use in order to prevent and treat alcohol use. Another important role of health services and health professionals is to inform the public about the harmful effects of alcohol on health and its social consequences and advocate social reactions in this regard. Increasing the capacity of health and social welfare systems is essential for the prevention, and treatment of alcohol-related disorders. Interventions for screening and brief interventions for dangerous and harmful drinks should be supported within the primary health care services. Integrated prevention, treatment, and care strategies should be developed for alcohol use disorders and concomitant conditions, including drug use disorders, depression, suicides, HIV/AIDS, and tuberculosis. There is no doubt that establishing and maintaining a recording and monitoring system for alcohol-related morbidity and mortality through regular reporting mechanisms will be an effective step in this regard.

Community action is one of the most frequently reported interventions to reduce the harmful use of alcohol. WHO recommends those governments and other stakeholders who support and strengthen civil society organizations to take joint action to reduce harmful alcohol consumption and alcohol-related harm. Rapid assessments need to be supported to identify gaps and priority areas for community-level interventions. Activating the community will be an effective way to prevent the sale and consumption of alcohol to younger users and provide and support alcohol-free environments for young people and other at-risk groups.

When the blood alcohol concentration (BAC) reaches a limit of 0.05%, sensory, motor and intellectual abilities of the drivers are impaired [24]. It is estimated that the highly visible, open public, and frequent use of checkpoints to reduce the use of drink-driving, may reduce fatal accidents by 20% [5]. WHO recommends the determination of the BAC limits to reduce the use of drink-driving, having checkpoints and random breath tests to reduce the use of alcohol and suspending the driver’s license if alcoholic users are detected. According to the 2016 Global Alcohol and Health Report, in most countries (70%) BAC is 0.05% or less. In 37 countries, the BAC limit was accepted as 0.08%, as the second most commonly used BAC limit. Fifteen countries reported the maximum allowed BAC at the national level to be 0 for drivers. In 31 countries, there is no BAC limit. While 80 countries (57%) stated that they had laws based on BAC values, 61 (43%) countries based their legislation on alcohol concentration values (alcohol blowing test) measured randomly by the police [5]. Fines are the most common method of alcohol penalty implemented by countries. Other common methods are the suspension of driving licenses and short-term detention. Additionally, zero tolerance towards novice drivers who drink and drive also mentioned as an effective method.

One of WHO’s best procurement policies in low- and middle-income countries are the practices that restrict physical access to alcohol. Increasing the price of alcohol is one of the most effective strategies to reduce the harmful use of alcohol. Heavy drinkers and consumers, including young people, are sensitive to changes in beverage prices. To regulate the hours, days and intensity of alcohol outlets and raising the national legal age limit for drinking and consuming alcohol is another effective strategy used to reduce alcohol use and harm. In countries that apply national legal age restrictions, the minimum age ranges from 13 to 25 years. Another important consideration is the danger of the development and increase of the illegal alcohol market in countries where physical access is restricted by very strict restrictions. Also, precautions should be taken for secondary alcohol use provided by parents and friends. Prevention of alcohol access to the young population is unfortunately not easy due to the increased sponsorship of alcohol brands for sports and cultural activities, increased communication techniques such as product placement, SMS, e-mail and social media, and the use of complex marketing techniques. It is possible that young people who start drinking alcohol at an early age will have more consumption in the coming years. In a qualitative study on alcohol control policies conducted by Mercen et al. in 2018, participants stated that bans would not be an effective alcohol control policy; on the contrary, they would make it interesting and people would reach alcoholic beverages in some way [7]. It is considered that it would be better to implement positive practices instead of prohibitions. In fact, increasing the opportunities for social activity and encouraging participation in social life covering the whole society, especially for young people at the age of onset is a necessity related to this issue.

WHO proposes the establishment of a local taxation system in its member countries. The existence of an illegal market for alcohol complicates policies on taxation in many countries. Unregistered alcohol includes unofficially produced alcohol and illegal alcohol. Informal and illegal alcohol production poses certain health and policy challenges. Illegal or informal consumption of alcohol may have additional adverse health consequences due to high ethanol content and contamination with toxic substances such as methanol. Unregistered alcohol may also contain potential contaminants, and can promote low-cost heavy drinking. The level of unregistered alcohol consumption is difficult to measure and this impedes countries’ efforts to tax, control, and produce legal alcohol. The most common methods of monitoring informal or illegal alcohol are police investigations, complaints systems, and case reports.

In conclusion; policies and measures to prevent alcohol use are not implemented adequately and alcohol-related disease burden continues to increase dramatically [10,21]. Public policies should be formulated with a public health perspective developed based on scientific findings to prevent and reduce the harmful effects of alcohol. Policies should be sensitive and fair to national, religious, and cultural contexts. The protection of people with high alcohol consumption and exposure to harmful effects should be an integral part of policies regarding the harmful use of alcohol. Public policies and interventions to prevent and reduce alcohol-related harm should cover all alcoholic beverages.

## Acknowledgment

No funding has been received for this paper.
